# The public attitude towards ChatGPT on reddit: A study based on unsupervised learning from sentiment analysis and topic modeling

**DOI:** 10.1371/journal.pone.0302502

**Published:** 2024-05-14

**Authors:** Zhaoxiang Xu, Qingguo Fang, Yanbo Huang, Mingjian Xie

**Affiliations:** 1 Department of Data Science, School of Computer Science and Engineering, Guangzhou Institute of Science and Technology, Guangzhou, Guangdong, China; 2 Department of Management, School of Business, Macau University of Science and Technology, Macao, China; 3 Data Science Research Center, Faculty of Innovation Engineering, Macau University of Science and Technology, Macao, China; 4 Department of Decision Sciences, School of Business, Macau University of Science and Technology, Macao, China; IBS Hyderabad: ICFAI Business School, INDIA

## Abstract

ChatGPT has demonstrated impressive abilities and impacted various aspects of human society since its creation, gaining widespread attention from different social spheres. This study aims to comprehensively assess public perception of ChatGPT on Reddit. The dataset was collected via Reddit, a social media platform, and includes 23,733 posts and comments related to ChatGPT. Firstly, to examine public attitudes, this study conducts content analysis utilizing topic modeling with the Latent Dirichlet Allocation (LDA) algorithm to extract pertinent topics. Furthermore, sentiment analysis categorizes user posts and comments as positive, negative, or neutral using Textblob and Vader in natural language processing. The result of topic modeling shows that seven topics regarding ChatGPT are identified, which can be grouped into three themes: user perception, technical methods, and impacts on society. Results from the sentiment analysis show that 61.6% of the posts and comments hold favorable opinions on ChatGPT. They emphasize ChatGPT’s ability to prompt and engage in natural conversations with users, without relying on complex natural language processing. It provides suggestions for ChatGPT developers to enhance its usability design and functionality. Meanwhile, stakeholders, including users, should comprehend the advantages and disadvantages of ChatGPT in human society to promote ethical and regulated implementation of the system.

## 1 Introduction

In such an era of rapid development of artificial intelligence (AI), ChatGPT has demonstrated remarkable capabilities and expanded in most life domains. Since it was introduced, with great potential applications in education, healthcare, industry, agriculture, travel, transportation, e-commerce, entertainment, marketing, and finance. The GPT (Generative Pre-trained Transformer) model represents a significant breakthrough in natural language processing, propelling the advancement of language-capable machines that resemble human communication [1]. ChatGPT is a specific application developed by OpenAI, a private artificial intelligence research lab, based on the GPT-3.5 model, released on November 30, 2022. The GPT-3.5 model is a technological iteration of the GPT-3 model. Based on the GPT-3.5 model, ChatGPT is trained on an extensive dataset comprising both text and code. It could generate text, perform language translation, produce various forms of creative content, and provide informative responses to user inquiries [2]. On March 15, 2023, OpenAI unveiled the new large-scale multimodal model, GPT-4, which processes textual data, incorporates image content, and exhibits improved response accuracy. Users can access GPT-4 through ChatGPT Plus on a fee-paying basis [2].

ChatGPT had over 100 million users in January 2023 [3]. The website generated 1.6 billion visits in June 2023 [4]. Mainstream media outlets have both expressed admiration and concern in response to ChatGPT. For example, the Guardian published an article written by GPT-3, asserting that humans should trust and respect AI’s role in improving their lives [5]. The New York Times published an article praising GPT-3’s impressive capabilities, noting its flaws [6]. In contrast, the Washington Post discussed the potential risks of AI, including ChatGPT, and argues for an enhanced regulatory framework in nations worldwide to address the issue urgently [7]. The public holds diverse opinions regarding significant new technologies [8], including ChatGPT. Some people believe that ChatGPT has many positive implications for society, such as helping people learn new knowledge and languages, generating creative text formats, replacing repetitive and laborious tasks, and providing companionship and support. Conversely, there are dissenting opinions regarding GPT, emphasizing its ongoing developmental stage, limitations, ethical issues, and possible outcomes of job losses.

Public attitudes toward ChatGPT are an important research topic. First, studying the public’s attitudes toward ChatGPT can help predict how widely ChatGPT will be used and accepted [9]. Public attitudes toward ChatGPT encompass affect, behavior, and cognition [10]. This study uses topic modeling and qualitative content analysis to examine how individuals perceive ChatGPT. The sentiment analysis of posts and comments allows this study to understand people’s emotions on ChatGPT. Second, public attitudes play an important role in shaping applied ethics. If the public is concerned about ChatGPT being used for malicious purposes, this could prompt a shift in its developmental trajectory and encourage its ethical utilization. Third, understanding public attitudes can lead to its advancement that better serves users. It provides researchers and practitioners related to AI with valuable insights into the potential applications and major limitations of ChatGPT. Understanding users’ concerns, needs, and preferences can help practitioners personalize ChatGPT’s responses to individual users and make ChatGPT more engaging and interactive.

This study aims to explore public attitudes toward ChatGPT, using data collected from users’ posts and comments on the social media site Reddit. A large number of studies have used data from Reddit to examine public perceptions, attitudes, and opinions. Reddit has approximately 57 million daily active users until 2023, one of the largest social media outlets in terms of users [11, 12]. Based on the data of posts and comments from Reddit, three research questions will be addressed:

RQ 1: What are the main topics on Reddit regarding ChatGPT?
RQ 2: What is the public sentiment toward ChatGPT?
RQ 3: How does public sentiment towards ChatGPT change over time?

The study employs the LDA model for topic modeling to identify emerging themes in public discussions about ChatGPT. In addition, sentiment classification is performed using a weighted combination of VADER and Textblob, and the results are presented in the form of bar charts and pie charts to analyze the variations in public sentiment towards ChatGPT. Furthermore, by extracting the creation timestamps of each sample and the sentiment score results, a daily change curve is plotted to illustrate the fluctuations in the number of positive and negative samples since 2023. This allows us to examine whether sentiment attitudes have changed over time.

This study would make the following contributions: First, this research represents a cutting-edge exploration of ChatGPT after other AI. As Artificial Intelligence advances, AI products, such as chatbots, smart virtual assistants, and self-driving cars, bring innovation, efficiency, and value creation to various fields. Therefore, extending the research line in AI studies, particularly focusing on ChatGPT, is crucial for understanding its impact, addressing challenges, fostering innovation, and ensuring the positive role of AI technology in society. Second, it seeks to address a research gap in public sentiment toward ChatGPT. Prior studies on ChatGPT have predominantly focused on its applications in education, academic research, tourism, and healthcare. Despite the widespread impact on human society, there is a lack of research regarding the public’s perspectives and sentiments regarding its influence. There are only a handful of exceptions wherein students were sampled as subjects to investigate attitudes toward the use of ChatGPT. However, there is a significant absence of comprehensive reviews that examine its implications on human society and public sentiment. This research provides insights into the public’s expectations and concerns regarding the potential risks and benefits of ChatGPT. Third, compared to traditional sampling surveys with self-reported questionnaires, social media big data analytics is leading-edge and efficient. Traditional public attitude surveys typically rely on questionnaires, which are time-consuming, costly, and limited to specific populations such as students, consumers, or employees. In contrast, social media data is publicly available and can be collected globally, allowing researchers to gather extensive data more quickly [13]. It facilitates comprehension of diverse perspectives on ChatGPT among various social groups. In addition, traditional fixed-choice questionnaires often only cover a limited number of questions [14]. Social media data provides in-depth information than structured questionnaires, as it offers insights into users’ interests, viewpoints, behaviors intentions on ChatGPT. Social media data could be real-time and quickly identify trends and patterns in public [15]. Social media big data analytics has been increasingly adopted in research on public sentiment towards AI products [16]. This study follows the current trend. Fourth, in practical terms, this research will provide valuable information for ChatGPT developers on enhancing the system to meet user needs better. By gathering user’s feedback on ChatGPT, the study will identify its strengths and weaknesses. It aids developers in improving ChatGPT’s functionality and performance, making it more user-friendly and ensuring an enhanced user experience.

## 2 Literature review

This section reviews the use of ChatGPT in human society for an initial assessment of the general public’s perception of ChatGPT. According to the bibliometric results, a total of 365 research papers (including articles and reviews) titled "ChatGPT" are found in the Web of Science database. Since ChatGPT was released in June 2020 based on GPT-3, the timeframe is set for the papers from June 1, 2020, to September 1, 2023. The existing research on the impact of ChatGPT on human society focuses on education, academic research, healthcare, tourism, and ethics. Education and healthcare are the fields with the highest number of published articles.

In education, research on the impact of ChatGPT covers medical education, nursing education, science education, language education, programming education, etc. [17]. ChatGPT offers opportunities for students and educators, including personalized feedback, increased accessibility, interactive dialogues, lesson planning, assessment, and helping students improve their programming skills. When ChatGPT is used to teach different subjects, varied pedagogical outcomes are achieved [18]. The use of ChatGPT for teaching mathematics, sports science, and psychology to students is unsatisfactory. Unexpectedly, ChatGPT has demonstrated reliability and utility, even in more rigorous medical education [19]. One of the most critical topics is the significant impact of ChatGPT on students’ programming learning [20]. A study on ChatGPT in computer programming learning proves its effectiveness and usability in generating solution code, checking bugs, debugging code, and dealing with programming assignments, exams, and homework [20, 21].

When exploring the impact of ChatGPT on academic research, academics usually focus on the following two questions: First, is the use of ChatGPT in a written work to be considered plagiarism? Second, can ChatGPT be considered as a co-author? There are polarised views on these two issues. Some positively embrace the new technology and see ChatGPT as a viable collaborator. In January 2023, Nurse Education in Practice, a journal published by Elsevier, generated significant controversy by acknowledging ChatGPT as a co-author [22]. However, others argue that using the ChatGPT technique constitutes academic cheating [23]. Many journals have stated that AI tools, such as ChatGPT, are not eligible to be credited as authors, including Science [24]. There is an undeniable consensus about ChatGPT as a competent co-author because of its ability to output more coherent, fairly accurate, informative, and systematic knowledge texts. At the same time, ChatGPT can support interdisciplinary research and provide research support [25]. The GPT model learns a large amount of textual data from different domains during training, giving it knowledge and understanding across multiple subject areas.

In healthcare, research indicates that ChatGPT holds enormous potential in virtual consultations, improving public mental health and well-being [26]. Moreover, studies have confirmed the positive role of ChatGPT in clinical practices and patient education [27]. Scholars have proposed various applications of AI in mental health, such as assisting clinicians with time-consuming tasks like documenting and updating medical records, enhancing diagnostic accuracy and prognosis, fostering a better understanding of mental illness mechanisms, and refining treatment through biological feedback. Furthermore, ChatGPT has even outperformed humans in emotional awareness evaluations. It is expected to assist physicians in making decisions related to diagnosing, treating, and managing chronic obstructive pulmonary disease [28]. Most healthcare researchers have expressed positive or balanced attitudes toward ChatGPT by analyzing data in social media [26]. These research findings collectively demonstrate the positive impact of ChatGPT and AI technologies on enhancing healthcare standards and patient experiences within the medical and healthcare domains.

Scholars have also shown significant interest in the impact of ChatGPT on tourism [29]. It is expected to bring about significant changes in the tourism industry by enhancing decision-making support for managers in tourism companies and policy-makers in governing bodies. The use of ChatGPT in tourism decision-making differs greatly from traditional approaches, as it engages tourists in an interactive question-and-answer mode. It allows them to personalize travel plans and recommend suitable travel services, including hotels, restaurants, transportation, local attractions, and leisure activities [30].

Scholars have also conducted exploratory research around ChatGPT in other fields. These include the following areas: corporate governance [31], supply chains [32], finance [33], intelligent vehicles [34], and so on. These researches demonstrate the prospect of wide application of GPT technology and lay the foundation for future research.

However, there have been some general complaints and ethical concerns regarding ChatGPT. First, criticism revolves around potential privacy leakage issues. This is because ChatGPT processes input prompts that may contain personal information, raising privacy concerns. Although OpenAI promises not to collect personal information from users, there is still a risk of leakage during network transmission due to inadequate security measures within data storage systems. Second, there are criticisms the use of ChatGPT in academia raises concerns about academic integrity. If ChatGPT fails to cite reference sources appropriately, it may lead to plagiarism or deception in education and academic research [35]. ChatGPT could be also used for online exams, posing a significant threat to exam integrity [36, 37]. To counter these problems, some anti-plagiarism techniques have been employed to detect AI-generated context [38]. To leverage ChatGPT’s advantages Responsibly in the realm of education, scholars suggest that educators should focus on improving students’ creativity and critical thinking rather than just acquiring skills. Meanwhile, AI-related tasks can engage students in solving real-world problems [39].

Third, the conversational capabilities of ChatGPT often draw criticism due to the limitations of its output. These limitations include inaccurate, fabricated, and biased information, along with a lack of in-depth understanding [39]. For example, ChatGPT does not have real-time information, and its training data comes from before September 2021, which could lead to biased responses. For example, using ChatGPT in medical research raises concerns about accuracy and reliability. The model has limitations in providing personalized advice and may sometimes generate inappropriate or outdated reference information. Due to a lack of human reasoning ability, ChatGPT may have difficulty generating responses to complex or abstract questions and understanding the context of text input [30]. In addition, ChatGPT struggles with identifying spelling errors, understanding colloquial and ambiguous language, and lack of interactive experiences and human emotions. Consequently, ChatGPT’s current capacity only enables it to partially substitute for human decision-making [40].

Fourth, some studies focus on the political issues raised by ChatGPT. Although ChatGPT often claims to be apolitical, empirical evidence demonstrates that it exhibits certain political predispositions, notably favoring supporting environmental protection and left-leaning liberal ideology [41, 42]. This may be because ChatGPT is trained on large text corpora collected from the Internet. These corpora may be dominated by influential institutions in Western society, such as mainstream news media, prestigious universities, and social media platforms. Consequently, these collections of texts may appear to represent a majority on certain topics. Furthermore, these algorithms may create an accumulation of false, inaccurate, biased, or confrontational content text on the web, exacerbating the vicious cycle of providing misleading and polarising information to the political system.

Research on the significant impact of ChatGPT in various domains of human society initially reflects stakeholders’ attitudes toward ChatGPT. Given the complexity of ChatGPT’s impact on human society, public opinions and attitudes toward ChatGPT also vary greatly, showing varying degrees of preference or aversion. However, limited literature comprehensively explores the public’s attitudes toward ChatGPT, and this study seeks to fill the gap.

## 3 Methods

This study aims to investigate public discourse and sentiment on ChatGPT through topic modeling and sentiment analysis using natural language processing based on the data from Reddit users’ posts and comments. The data science methods provided an efficient way to classify latent topics and sentiments in public discourse. Firstly, word frequency reveals the public’s interests related to ChatGPT. Word cloud visualization intuitively presents these high-frequency terms, making critical information easily accessible [43]. Secondly, topic modeling facilitates the identification of latent topics within textual data, which are more comprehensible to interpret [44]. Public discussions are often multifaceted and different words and sentences may relate to the same or interconnected topics. Topic modeling helps cluster related content, leading to a better understanding of the associations among various subjects [45]. This is crucial for exploring the breadth and depth of discussions from diverse perspectives. Lastly, sentiment analysis enables the identification of emotional tendencies (positive, neutral, or negative) conveyed within the text [46, 47]. During public discussions about ChatGPT on Reddit, sentiment analysis aids in gauging public sentiment, revealing positive attitudes, concerns, and potential issues or needs. Analyzing sentiment fluctuations over time tracks emotional shifts helps to comprehend the public’s responses to specific events, such as ChatGPT product releases, or promotions. Through this approach, emotional changes and factors can be uncovered.

### 3.1 Data collection and cleaning

Social media platforms such as Twitter, Facebook, Instagram, and Reddit allow users to express their emotions, interests, hobbies, and opinions in real-time within an online community. Reddit (https://Reddit.com) has approximately 57 million daily active users until 2023, one of the largest social media outlets in terms of users [11]. Reddit users can share text, links, images, or videos in various sub-communities (called subreddits and dedicated to specific topics) [48]. Everyone has access to the public subforum (called subreddits on Reddit), and users can comment and vote on posts and comments for free and anonymously. Reddit has more than 100,000 active subreddits as of 2023. A large number of existing studies use data from the platform to examine public perceptions, attitudes, and opinions. These studies cover a broad range of topics, including disasters [49], vaccines [50], advertisement [51], tobacco [52], vehicles [53], climate change [54], digital governance [55], political psychology [56] and collective identity [57]. The Application Programming Interface (API) is a set of tools that defines how a software application interacts with other components, services, or platforms [58]. APIs allow for communication and data exchange between different software systems, enabling them to connect and interoperate with each other.

In terms of ethical considerations in the present research, the official API of Reddit is freely and publicly available to third parties [12, 59]. In accordance with Reddit’s privacy policy, developers are permitted to write programs or applications, such as Apify used in this study, that interact with the Reddit platform through specific requests and commands. These actions include retrieving specific information, posting content, or performing other tasks [60]. Reddit enables researchers to extract the subreddits, threads, comments, and associated metadata through various programming languages [61]. Therefore, data collection and analysis in this study comply with the terms and conditions of the data source. Following the principle of data minimization, it only collects data relevant to the purpose of the study. In addition, to adhere to the principles of privacy and untraceability, personal user information is anonymized because Reddit posts and comments are user-generated.

This study utilized Apify (https://apify.com) for collecting Reddit posts and comments as the primary data source. Apify is a platform for data collection for web scraping, data extraction, and automation. It offers tools and services that assist developers in extracting data from web pages, executing automated tasks, and building web crawlers. Personal information has been anonymized. The data consists of posts and comments. The sample data is shown in Table 1.

**Table 1:** An example of a sample post regarding GPT-3.

"id": "**********",
"parsedId": "14a695s",
"url": "https://www.Reddit.com/r/GPT3/comments/14a695s/what_next/",
"username": "AutoModerator",
"title": "What Next?",
"community name": "r/GPT3",
"parsedCommunityName": "GPT3",
"body": "So, we recently locked down for two days in protest of Reddit’s API changes…",
"numberOfComments": 54,
"upVotes": 17,
"isVideo": false,
"isAd": false,
"over18": false,
"createdAt": "2023-06-15T16:05:21.000Z",
"scrapedAt": "2023-08-15T06:03:56.688Z",
"dataType": "post"

It sets GPT3, GPT3.5, and GPT4 as keywords. These keywords aim to examine whether people’s perceptions of ChatGPT have evolved, particularly since the ChatGPT update. Apify collected 11,730, 10,109, and 12,046 relevant entries (posts and comments) for the respective keywords. This study performed random sampling to ensure sample uniformity, resulting in 10,000 entries preserved for each GPT version. The dataset consisted of 30,000 samples from June 2020 to August 15, 2023.

In terms of data cleaning, emoticons, digits, punctuation, links, unnecessary words, non-ASCII characters, and stopwords were removed from the textual data suggested by Yadav et al [61, 62]. The NLTK library provides the English stopwords list. It consists of common English words with no semantic or informational value [63, 64]. Stopwords are typically filtered out in natural language processing to enhance the efficiency and accuracy of text analysis. Additionally, all uppercase letters have been converted to lowercase. Furthermore, considering a slight overlap in the data sources, this study also conducted duplicate text filtering. In the end, 23,773 entries were retained, forming the database Processed_GPT_total.json, displayed in supporting information.

To improve the reproducibility of our research results, the data and research design have been stored on Protocols.io. Protocols.io has assigned a protocol identifier (DOI) to our protocols, which is DOI: dx.doi.org/10.17504/protocols.io.bp2l6xee1lqe/v1. Open Access license is freely available for anyone. Readers can find relevant details by visiting the following URL: https://www.protocols.io/private/899FA95EAEFD11EEAB870A58A9FEAC02.

### 3.2 Topic modeling

All data analyses were processed using Python (version 3.10). For topic modeling, prior research proposes three techniques: Latent Dirichlet Allocation (LDA), which adeptly discerns latent topics through probabilistic approaches; Non-Negative Matrix Factorization (NMF), which underscores the intricate relationship between documents and their inherent topics; and Transformer-based models, renowned for their proficiency in grasping the intricate semantics embedded within textual data [65].

In this study, the Latent Dirichlet Allocation (LDA) model will be used to conduct the topic model. It is a hierarchical Bayesian probability model consisting of three levels: documents, topics, and words. The fundamental concept behind the LDA model is that each document can be represented as a mixture distribution of latent topics, where each topic is a probability distribution over all words in the vocabulary [66]. The LDA model introduces latent random variables, specifically the topic mixture weights θ, which are T-dimensional parameters representing the topics. These topic mixture weights are endowed with a Dirichlet prior distribution [67].

$$Dir(\alpha_{1}\cdots ,\alpha_{r})=\frac{\Gamma \left(\sum_{j}\alpha_{j}\right)}{\prod_{j}\Gamma (\alpha_{j})}\prod_{j=1}^{T}\theta_{j}^{\alpha_{i}-1}$$ (1)

The parameters α_1_,…, α_T_ in the Dirichlet distribution are regarded as hyperparameters for the multinomial θ = P(θ_1_,…, θ_T_). Each hyperparameter αⱼ can be interpreted as a prior distribution on the frequency of sampling topic j from a document. To facilitate computation and due to the assumption that different topics are used similarly across documents, LDA assumes the exchangeability of topics within a document. For computational convenience, the model assumes symmetric Dirichlet distributions with identical hyperparameters α, i.e., α₁ = α₂ =… = α_T_ = α. It is worth noting that Dirichlet distributions and multinomial distributions are conjugate distributions. Assuming a T-dimensional random vector θ follows a Dirichlet distribution, the T components θ₁, θ₂,…, θ_T_ are continuous and non-negative, satisfying θ₁ + θ₂ +… + θ_T_ = 1. In the initial LDA literature, Dirichlet priors were mainly introduced on the document-topic distribution θ, without assuming priors on the topic-word distribution φ. However, for leveraging the properties of conjugate distributions and facilitating inference and computation, subsequent research introduced a Dirichlet before the topic-word distribution φ, assuming the exchangeability of words within a topic. The hyperparameter of this Dirichlet prior is denoted a s β. Consequently, the LDA model incorporates two sets of priors: one for the document-topic distribution from a symmetric Dirichlet distribution (α) and another for the topic-word distribution from a symmetric Dirichlet distribution (β) [68].

Perplexity is a common metric used to evaluate the performance of topic models and is particularly applicable to a selected number of LDA topics to model. It measures how well the model fits the documents in a given dataset, with a lower perplexity score indicating better model performance on that dataset.

The perplexity is calculated based on the likelihood probability of the model on the training set by computing the average log-likelihood probability on the word sequence of each document on the training set. Perplexity is calculated through the Equation in LDA:

$$Perplexity(W)=exp\left\{-\frac{\sum_{d=1}^{M}\sum_{w_{i}\in d}log(\sum_{p\in K}p(z_{p}|d_{j})P(w_{i}|z_{p}))}{\sum_{d=1}^{M}N_{d}}\right\}$$ (2)

Where *N*_*d*_ is the number of words, and *M* is the size of the document *d*. *N* is the size of the words set, and *K* is the size of the topic set. *p*(*z*_*p*_|*d*_*j*_) is the probability that the topic *z*_*p*_ appears in document *d*_*j*_. *P*(*w*_*i*_|*z*_*p*_) is the probability that the word *w*_*i*_ occurs in *z*_*p*_. It is clear that perplexity is mainly influenced by *M*, *N* and *K* according to equation [69]. By comparing the perplexity scores for different numbers of topics, the optimal number of LDA topics to model can be determined to obtain the best fit on a given dataset, while also being mindful of overfitting issues [70].

### 3.3 Sentiment analysis

Existing research typically uses three approaches for sentiment analysis: rule-based approaches, machine learning, and deep learning [71]. Rule-based methods, often referred to as rule-based sentiment analysis, utilize predefined sentiment lexicons and rules to evaluate sentiment [72]. Machine learning methods, on the other hand, learn sentiment classification models from data through supervised or unsupervised learning, offering good generalization capability [73]. Deep learning methods such as RNNs, CNNs, and Transformers automatically learn features from text, adept at capturing long-term dependencies, albeit requiring substantial data and computational resources [74].

The present study employs a rule-based approach to classify sentiments in posts and comments. The sentiment categorization utilizes a weighted approach that combines VADER with TextBlob. By combining the strengths of TextBlob and VADER, two distinct sentiment analysis tools that employ different algorithms and semantic processing approaches, and weighting their results, the overall performance of sentiment analysis can be effectively enhanced. These tools exhibit varied performances in different contexts, and through weighting their outcomes, one can leverage their respective strengths to improve the overall performance of sentiment analysis. Simultaneously, this integrated approach helps mitigate individual biases of different sentiment analysis tools, enabling the system to better adapt to diverse text samples, especially those of specific types. Furthermore, when confronted with complex and diverse language expressions, a single sentiment analysis method may exhibit instability. Integrating multiple methods enhances the model’s robustness, allowing it to adapt to various types and styles of text flexibly. Such a comprehensive approach demonstrates significant advantages in improving the accuracy and adaptability of sentiment analysis.

The VADER sentiment analysis hinges on a lexicon that links linguistic components to emotional strengths, referred to as sentiment scores [75]. Calculating the sentiment score for a specific text is a straightforward process of summing up the intensity values for each word in the text. Notably, each word’s intensity falls within a range of -4 to 4, and this lexicon is derived from human evaluations. The sentiment score for a sentence is determined by adding up the sentiment scores of its sentiment-carrying words [76]. Nevertheless, it employs the Hutto normalization process on the final sum to bring it within a range of -1 to 1. The compound score is formulated as follows:

$$Compound\;Score=\frac{x}{\sqrt{x^{2}+\alpha }}$$ (3)

Where x is the sum of the sentiment scores of the constituent words within the sentence, and *alpha is* the normalization parameter. These scores aid in determining the polarity of a given sentence. It offers versatility in handling intricate textual data analysis tasks. Upon analyzing a sentence, it provides two outputs: polarity and subjectivity. Polarity yields a value within the range of [–1, 1], where -1 indicates a negative sentiment, and +1 signifies a positive sentiment. Conversely, subjectivity produces an output between [0, 1] and reflects personal opinions and judgments.

Rule-based sentiment analysis in TextBlob relies on natural language processing techniques, utilizing predefined rules and syntactic structures to identify the emotional polarity within the text [77]. Initially, the text is decomposed into words and phrases, and part-of-speech tagging is conducted to comprehend the grammatical roles of each word in the sentence. Subsequently, based on a pre-defined sentiment lexicon, each word is assigned a sentiment polarity score, such as positive, negative, or neutral. Rules may also take into account relationships between words, where the presence of negation words, for instance, could alter the emotional polarity. By weighting or averaging sentiment scores for all words in the text, the overall emotional polarity of the text can be determined. The advantage of this method lies in its simplicity and ease of implementation, while its accuracy can be enhanced by continuously updating and expanding the sentiment lexicon [78].

Two sentiment analysis tools are used in this study: TextBlob and VADER. Each tool possesses analytical strengths, making them invaluable for nuanced sentiment extraction [79]. TextBlob, with its linguistic expertise, provides a polarity score, while VADER, adept at discerning both context and sentiment intensity, yields a compound score. Given the distinct virtues of each tool, relying solely on one would be an oversight. Therefore, a composite sentiment score is meticulously derived from both [78]. Let *S*_*t*_ represent the sentiment score from TextBlob and *S*_*v*_ the score from VADER. If this study designates and as their respective weights such that a + b = 1, the weighted sentiment score, *S*_*w*_, is defined as

$$S_{w}=a\cdot S_{t}+b\cdot S_{v}$$ (4)

In this study, the weights are set at a = 0.4 for TextBlob and b = 0.6 for VADER, reflecting a slight inclination towards the latter’s comprehensive capabilities.

### 3.4 Sentiment trend analysis

Sentiment trend analysis, a burgeoning field, has become vital for comprehending public perception of specific issues [80]. In this study, sentiment trend analysis integrates the strengths of the aforementioned two methods, utilizing the results with a weighted approach. Initially, a DataFrame (df) is created to organize the data, encompassing columns for date, weighted sentiment scores, and sentiment labels (’Positive’ or ’Negative’). The date column undergoes conversion to the datetime type for accurate time series analysis. Subsequently, the ’Sentiment’ column values are determined based on the weighted scores, with ’Positive’ assigned if the score exceeds 0, and ’Negative’ otherwise. Finally, the DataFrame is grouped by date and sentiment, daily sentiment counts are computed, and a line plot is generated using matplotlib to illustrate the daily counts of positive and negative sentiments over time.

A lucid visualization is paramount to fully capturing the chronology of sentiment dynamics [81]. As such, daily sentiment metrics are illustrated, clearly depicting the populace’s emotional ebbs and flows. This graphical elucidation not only bestows a daily sentiment snapshot but also illuminates prevailing trends, proving indispensable for decision-makers, ranging from corporate strategists to policymakers, who anchor their choices on the pulse of public sentiment.

## 4 Result

### 4.1 Word frequency

**Fig 1:**
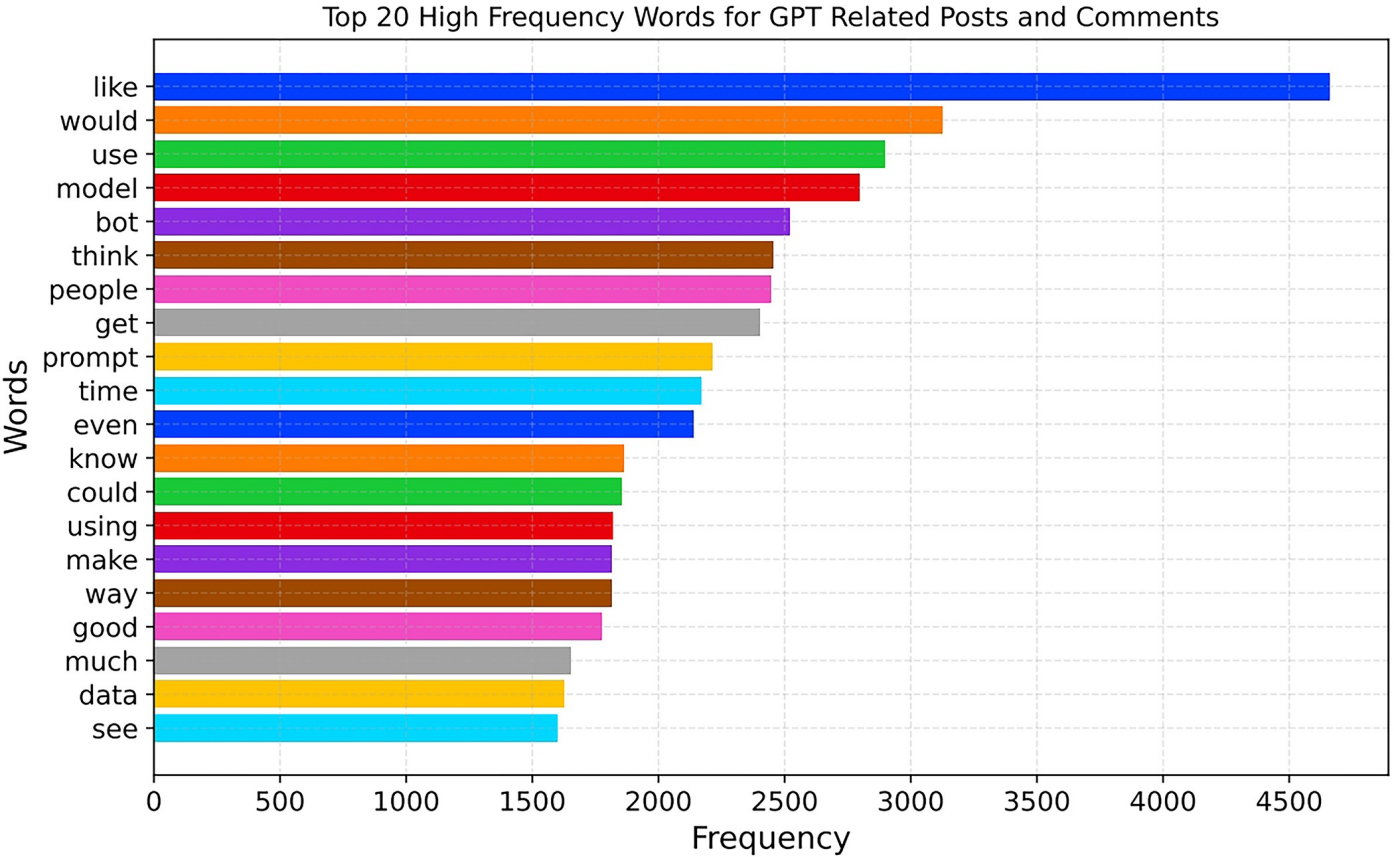
Top 20 most common words.

In this part, word clouds and frequency graphs provide initial insights into the diverse perspectives of the general public on the topics (Question 1) and attitudes (Question 2) toward GPT. The analyzed entries exceeded a total count of 23,773 entries. Fig 1 displays the top 20 most common words from the entries. As Fig 1 shows, the public’s positive attitude toward ChatGPT is evident through the words "like" and "good", indicating their appreciation and approval of the model. However, the discussions also unveil contemplation about the practical applications of ChatGPT, encompassing terms such as "use," "using," "way," "make," and "need," highlighting the discourse on how to harness ChatGPT’s capabilities fully. In addition, words like "would," "think," "know," "could," and "even," express doubts and uncertainties, reflecting concerns about its potential limitations and abilities. Technical aspects of the discourse include terms like "model," "bot," "prompt," "data," "code," and "models," revealing the audience’s attention to ChatGPT’s internal working model, data processing, and technological implementation.

### 4.2 Topic modeling

**Fig 2:**
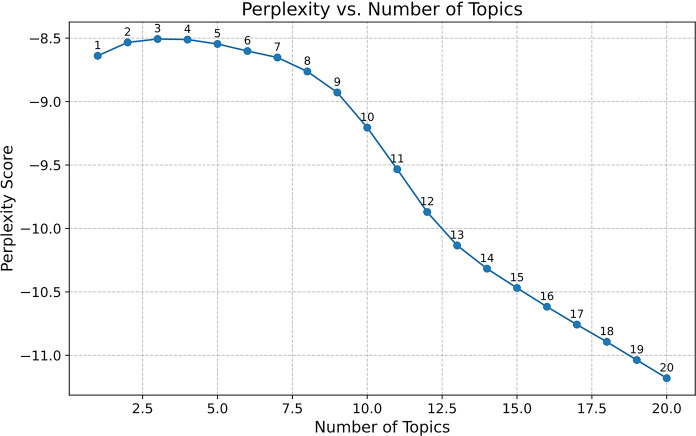
Perplexity vs. number of topics.

This part presents the emergent topics and themes identified through topic modeling. It aims to address research question 1: What are the emerging topics related to ChatGPT? This study combines qualitative and quantitative content analysis to uncover and discover latent topics and themes of the public’s discussion, which are believed to hold significant potential for research in the field of social media [82]. As a commonly used quantitative method for topic classification, the LDA model aids in determining the most optimal number of topics for classification. In Fig 2, the perplexity-topic number curve is plotted.

Typically, the optimal number of topics is determined based on lower perplexity levels [83]. When the number of topics is set at 8, the perplexity is at its lowest. However, the lowest perplexity may not always signify the best model performance. With a high number of topics, models often overfit, resulting in excessive and non-convergent topic counts. An excessive number of topics may lead to high redundancy, resulting in low distinctiveness and uniqueness between topics [71, 83]. Hence, many studies rely on human judges to determine the optimal number of topics. This method also adheres to certain principles: (1) high coherence between words and topics; (2) the quality of topic, ensuring non-repetition, non-conflict, and coverage of primary content [84]. This study tests the topic categorization and high-frequency words of each topic when the number of topics was set at 8 (Table 2). However, it demonstrates poor coherence and topic quality. Across the topics, there is a lack of coherent themes, with words appearing disjointed and unrelated within each category. The representative words fail to form distinct and meaningful topics, undermining the effectiveness of the model in capturing the underlying structure of the data.

**Table 2:** Top words of eight topics.

Topic	Representative words
1	great, use, chat, also, would, even, like, get, work, could
2	god, like, would, lol, got, time, still, yet, said, yes
3	like, data, would, training, something, words, word, language, even, still
4	answer, question, would, today, apples, man, time, asked, give, many
5	bot, please, open, image, prompt, free, openai, source, message, wait
6	people, like, think, know, get, even, human, want, make, much
7	api, access, app, use, pay, key, version, keyboard, subscription, free
8	code, write, use, writing, amp, ask, going, like, make, etc

Therefore, this study also tested the number of topics corresponding to the point of significant decrease in perplexity, i.e., the number of topics (7) near the inflection point of the curve. When the number of topics is set at 7, the distribution of word frequencies in relevant topics is shown in Table 3. The top words in each topic exhibit good coherence and topic quality. Table 3 shows that the top 10 words in each topic are categorized into seven topics, which are then assigned to three themes. The analysis of seven topics demonstrates the wide range of discussions regarding ChatGPT on the Reddit community. These discussions cover technical inquiries, philosophical pondering, impacts on society, creative applications, and entertainment. The topics reflect the multifaceted nature of ChatGPT and highlight the diverse perspectives and interests of the public when using it.

**Table 3:** Top words of seven topics and three themes.

Topic	Representative words	Theme
1	like, people, think, would, get, even, use, time, much, good	User Perception
2	bot, please, prompt, link, open, questions, amp, message, action, free	Technical Methods
3	consciousness, conscious, agi, humans, brain, god, human, believe, intelligence, self	User Perception
4	like, code, use, would, prompt, text, data, language, also, make	Technical Methods
5	man, music, art, state, top, health, home, quantum, menu, fire	Impacts on Society
6	amp, world, market, people, life, human, game, new, jobs, impact	Impacts on Society
7	gif, admit, gypsy, wide, care, forgot, trump, president, capital, spider	Impacts on Society

The first topic concerns people’s general impressions of ChatGPT. The keywords such as "like," "think," and "good" indicate that individuals are generally favorable towards ChatGPT. This topic focuses on how people perceive ChatGPT’s potential benefits, usability, functionality, and positive impact on them. The second topic appears to focus on technical inquiries for assistance. Terms such as "bot," "prompt," and "link" indicate that users are seeking information on how to use ChatGPT for various tasks. The terms "questions," "message," and "action" suggest a desire to optimize ChatGPT’s functionality for specific purposes. The third topic delves into philosophical discussions, examining consciousness, AGI (Artificial General Intelligence), and human reasoning of ChatGPT. The keywords like "consciousness," "agi," "humans," and "belief" imply that users are exploring ChatGPT’s human consciousness, intelligence, and spiritual characteristics. The fourth topic explores the technical details of ChatGPT, focusing on coding and textual manipulation. The keywords such as "code," "prompt," "text," and "language" suggest discussions on how to utilize ChatGPT for code generation or text creation efficiently. This topic covers its capabilities in software development, content creation, and language-oriented tasks. The fifth topic explores ChatGPT’s impact on diverse fields of life, including the arts, healthcare, and quantum phenomena. The words "music," "art," "health," and "quantum" indicate discussions on how ChatGPT brings the revolution and advances to the artistic and scientific domains.

The sixth topic focuses on the broad social and economic influences of ChatGPT. Keywords like "market," "jobs," and "impact" suggest discussions on ChatGPT’s impact on the job market and the global economy. The debates could probably revolve around potential job loss resulting from ChatGPT and the ethical concerns about AI. The seventh topic concerns the correlation between ChatGPT and politics and entertainment. The keywords such as "trump" and "president" relate to ChatGPT’s function in political discussions. The terms "spider," "gif," and "gypsy" indicate the potential utilization of ChatGPT within cultural and entertainment contexts.

Through the qualitative content analysis, seven topics were systematically coded and categorized into three themes. Theme 1 covers Topics 1 and 3, which focus on users’ positive views of ChatGPT and its potential advantages and positive influence on various aspects of life. Theme 2 encompasses Topics 2 and 4, focusing on the technical methods of ChatGPT, including queries, assistance, coding, and practical applications. Discussions cover topics such as the application of ChatGPT for specific tasks, seeking guidance, and sharing experiences regarding coding and language generation. Theme 3 comprises Topics 5, 6, and 7, focusing on the broader social impact of ChatGPT on art, music, health, politics, market, employment prospects, scientific progress, and the entertainment industry.

### 4.3 Robustness of topic modeling

To verify the robustness of the model, it randomly selects 10,000 samples as a subset from the original dataset for testing [85]. To examine the robustness, the same parameters are used for two models [86].

**Fig 3:**
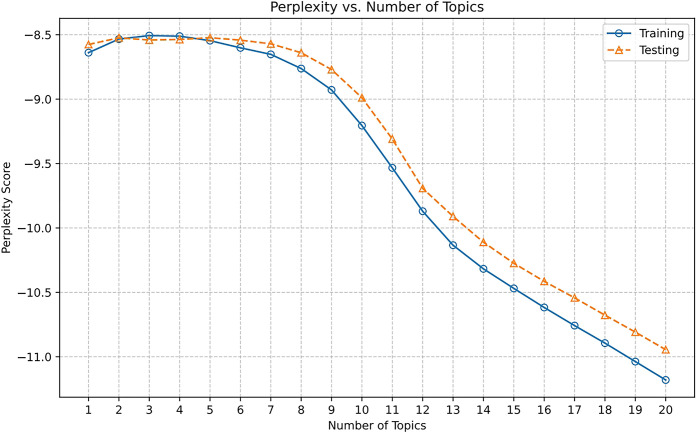
Robustness test-perplexity similarity test.

Fig 3, compares the perplexity scores of the two models when the number of topics ranges from 1 to 20, finding that the results of the two runs are highly similar. The solid line with circles represents the perplexity changes of the original dataset when LDA topic modeling is performed, while the dotted line with triangles represents the perplexity changes of a subset of the original dataset under the same modeling process. It can be seen that the variation range of the perplexity scores of the two curves is between -8.5 and -11.1, and the overall trend shows a slow increase at first and then a decrease. Within the topics range of 1 to 8, the difference between the two curves is minimal, showing a high degree of similarity. The largest gap is when the number of topics is 20, and it is only 0.23 at this time, indicating that our model is more robust.

When the topic modeling of the subset is performed and the number of topics is 7, the high-frequency words corresponding to each topic are shown in Table 4. Three Themes were summarized from the 7 Topics generated by LDA topic modeling based on a subset of the original dataset, which is consistent with our previous LDA modeling results using the original dataset. This also confirms the posts and comments about GPT. The three widely discussed themes are User Perception, Technical Methods, and Impacts on Society.

**Table 4:** Top words of seven topics and themes based on a subset.

Topic	Representative words	Theme
1	art, people, life, industry, market, pig, sister, business, technology, profit	Impacts on Society
2	use, api, code, prompt, openai, text, get, chat, content, version	Technical Methods
3	tom, youtube, also, tone, blog, words, use, copyright, pay, battery	Impacts on Society
4	like, would, know, good, get, people, really, time, even, something	User Perception
5	bot, prompt, please, open, questions, discord, automatically, free, subreddit, message	Technical Methods
6	houses, link, district, red, para, winter, governed, blue, sol, fewer	Impacts on Society
7	think, human, could, would, people, even, time, make, like, way	User Perception

**Fig 4:**
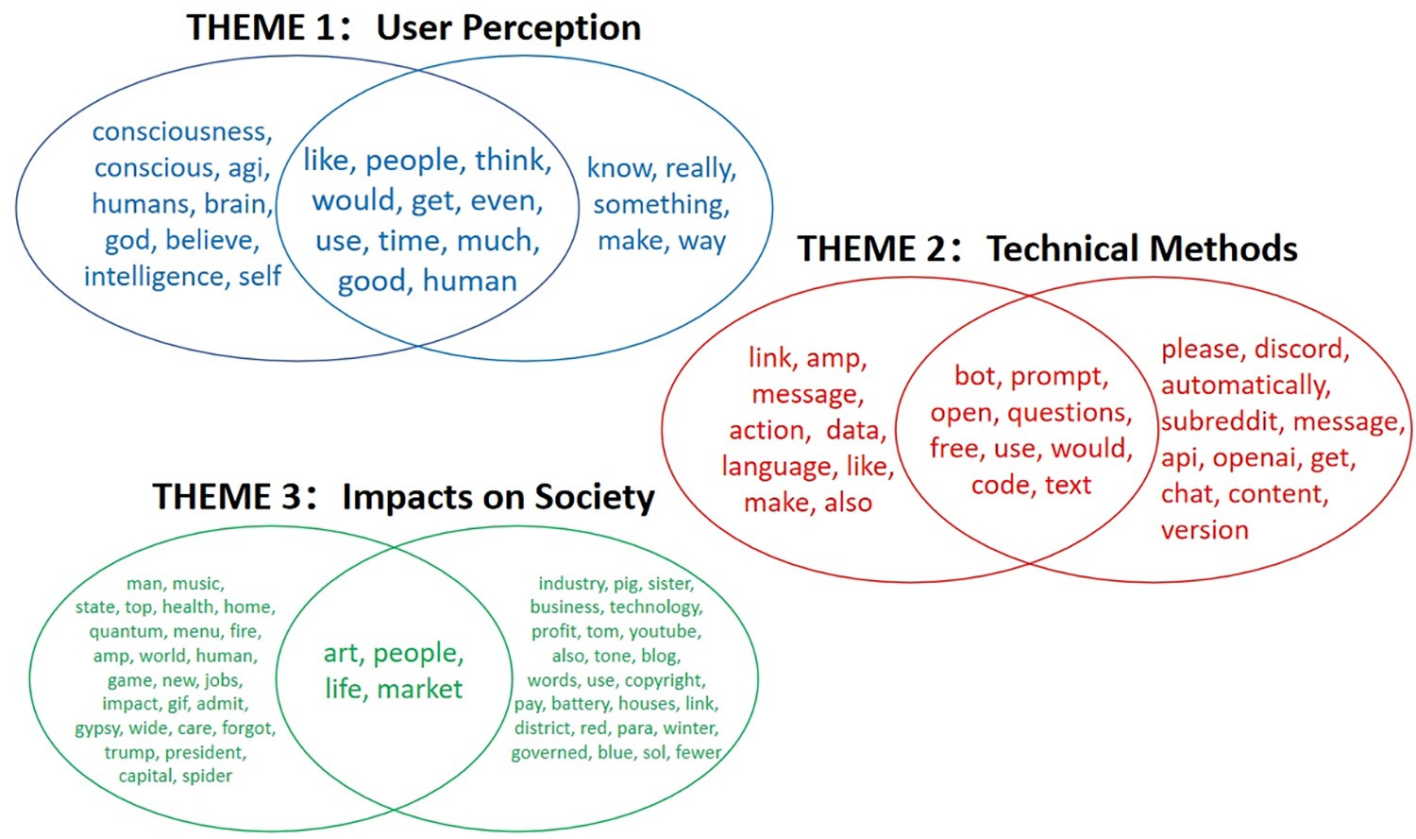
Theme similarity with the high-frequency word map.

To illustrate the robustness, the theme generated by the two models has a high degree of similarity with the top words (Fig 4). Regardless of whether it is applied to the original dataset or its subset, it can produce highly similar theme results. It has summarized the seven topics derived from the original dataset into three themes, and it also depicts the high-frequency words associated with different themes generated by two topic modeling processes. Among these, the intersection of each pair of themes represents the high-frequency words that yield identical results from both topic modeling techniques.

**Fig 5:**
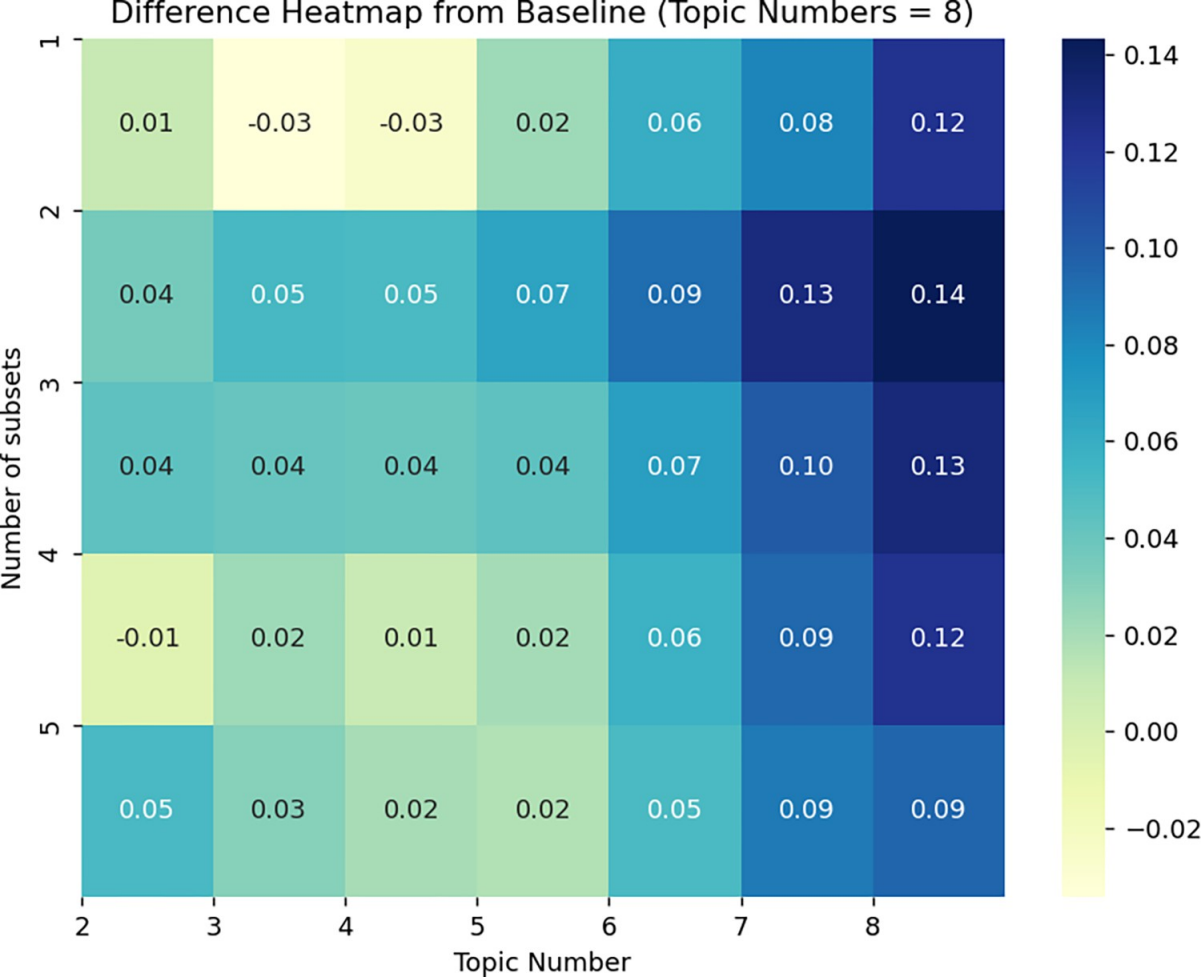
Perplexity difference heat map.

To further corroborate the reliability of this result, it repeatedly experimented with and sampled the original dataset five times [87]. Since comparing the results when the number of topics is 1 is not meaningful, it utilizes the perplexity scores from the second to the eighth topics of the original dataset as a baseline. These baseline scores are then compared against the perplexity scores generated by the subsets sampled on the other five occasions. A similarity test is subsequently conducted to assess the comparability shown in Fig 5. The horizontal axis of the heat map above represents the number of topics using LDA for topic modeling, ranging from 2 to 8, and the vertical axis represents the number of subsets we randomly extracted from the original dataset [88]. Different squares represent the difference in perplexity between the topic modeling results of 5 randomly selected subsets of the original data set and the topic modeling of the original data set.

In Fig 5, the difference between the five tests and the baseline (perplexity of the original dataset) is small. When the number of topics is less than or equal to 6, the difference in confusion does not exceed 0.10. The relative maximum difference is when the number of topics in the second extracted subset is 8, at which time the difference in perplexity is only about 0.14. After several times of randomly extracting the subset and re-performing the number of topics-perplexity experiment, the perplexity difference is very small for five experiments in the range of the number of topics is 8, which further confirms the high robustness of our modeling. Rre-performing topic modeling on randomly selected subsets and topic modeling performed on the original dataset show extremely high similarity in results, and the results of multiple repetitions of the modeling show very little difference in perplexity. Therefore, based on the comparatively low perplexity differences between the subsets and the original data, the robustness of the model can be supported.

### 4.4 Sentiment analysis

**Fig 6:**
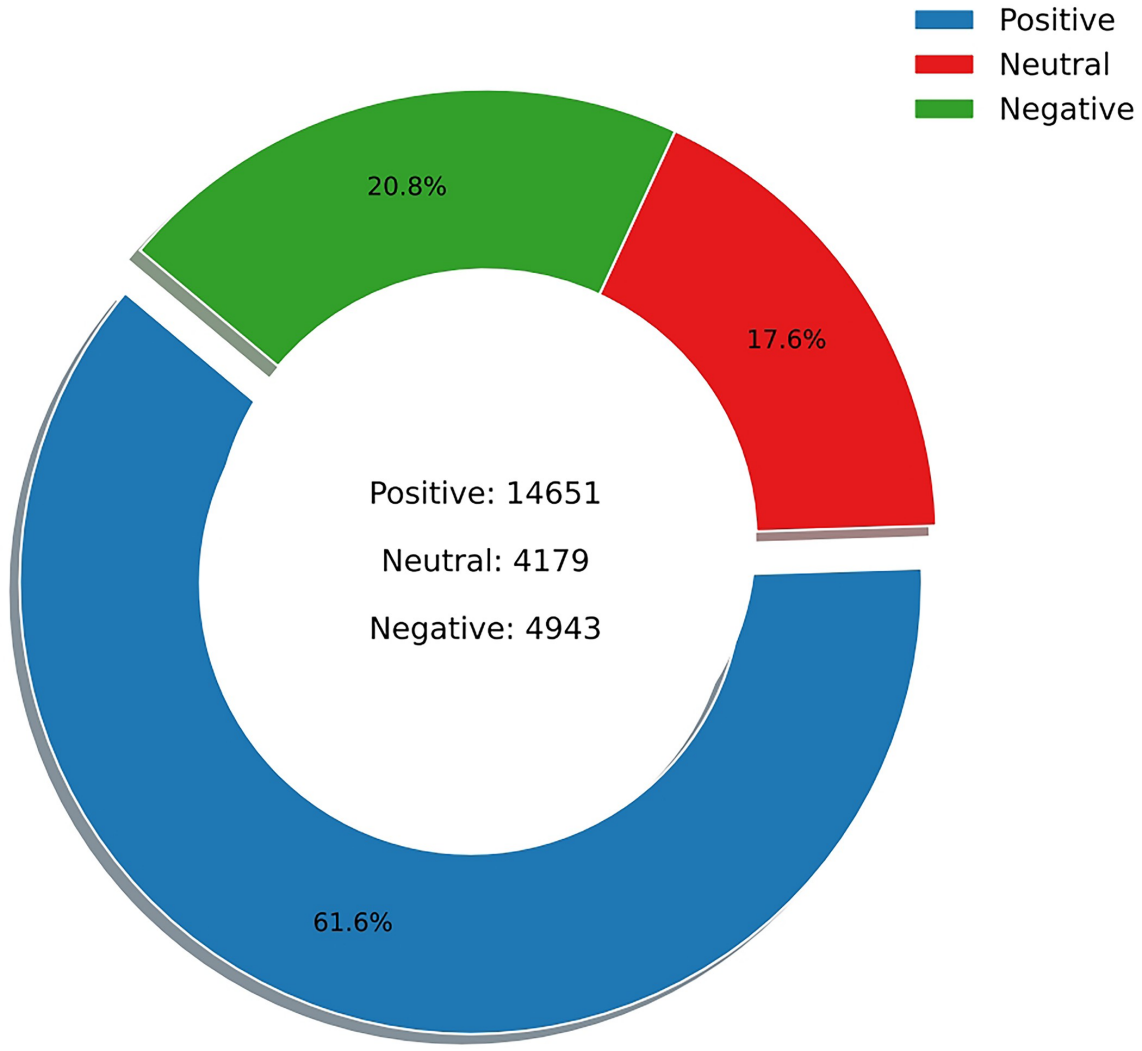
The weighting of the sentiment pie chart.

In this part, two sentiment analysis models, Vader and Textblob, are assigned weights of 0.6 and 0.4 respectively for sentiment classification to explore research question 2. The sentiment analysis categorizes the emotional tone of the entries into three distinct parameters: positive, negative, and neutral. The weighting of positive, neutral, and negative entries is shown in Fig 6 (N = 23,773). The analysis reveals a positive sentiment among Reddit users, with approximately 61.6% of entries conveying affirmative emotional nuances. In contrast, about 20.8% of entries express negative sentiments, while neutral ones account for the smallest segment at 17.6%.

**Fig 7:**
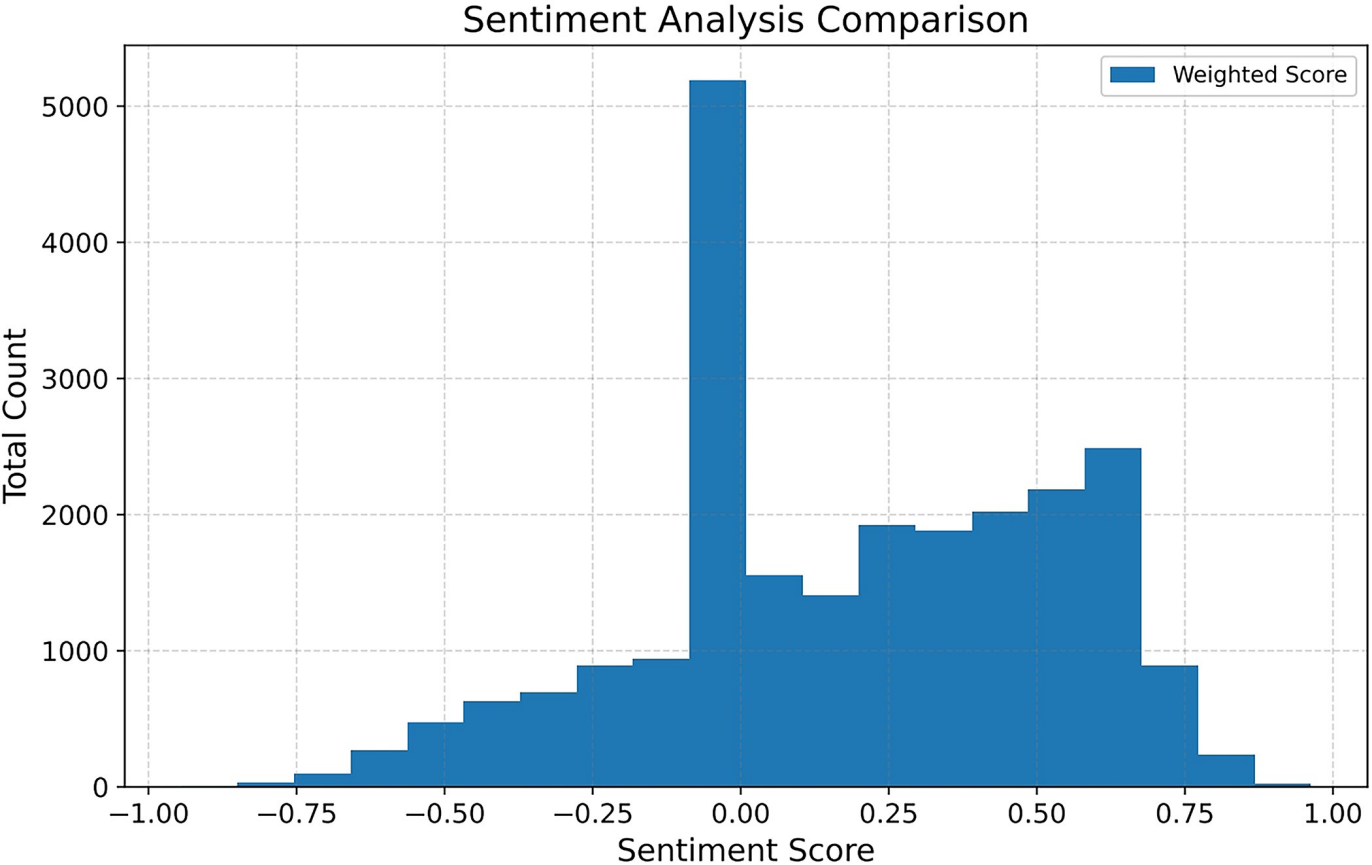
Sentiment distribution bar chart.

Fig 7 demonstrates the comprehensive sentiment distribution (N = 23,773). There is a noticeable concentration of entries between 0 and 0.6, indicating the prevailing positive emotions. Moreover, it is pertinent to mention that the sentiment analysis identified a significant count exceeding 5100 entries conveying a neutral sentiment. Furthermore, most entries fall within the range of -0.25 to 0.60, suggesting a moderately nuanced sentiment orientation and a notable absence of distinct polarization in the overall sentiment attitudes towards ChatGPT.

Table 5 selectively displays the high-frequency words in posts and comments expressing different sentiments.

**Table 5:** High-frequency words for entries with different sentiments.

Positive		Neutral		Negative	
Example	Frequency	Example	Frequency	Example	Frequency
bot	2385	model	364	time	336
model	2140	api	268	model	293
good	1540	prompt	258	way	254
way	1325	data	248	bad	247
better	1249	way	232	new	235
openai	1190	chat	225	wrong	230
data	1183	work	217	problem	221
new	1176	right	205	code	214
work	1146	code	200	work	197
open	1130	much	191	data	192

In neutral discussions, individuals mention the usage of ChatGPT, such as "api", and "to access it you have to use the API." The API is an interface that facilitates communication between distinct software systems or services, enabling programs or applications to access the functionalities or data of other systems. Users might engage in conversations regarding the functionalities and limitations of the API, deliberating on the prospect of integrating ChatGPT’s language generation capabilities into their applications or systems using the API [89].

The negative comments with words such as "wrong," "bad," and "problem," reflect their perception of errors, issues, or flaws of ChatGPT. For instance, "It is just a dumb stunt for a dumb application," "The model has been quantized badly, " and "I have doubts about their security claims. " These express suspicion about certain aspects of ChatGPT which generate problematic content at times or unsatisfactory functionality. It suggests skepticism about the accuracy and quality of the content generated by ChatGPT.

It is worth noting that the term "model" appears in both positive, neutral, and negative posts and comments, showcasing varying perspectives on the ChatGPT based on its performance, applications, and potential risks. Positive comments emphasize its impressive capabilities, including generating high-quality text, conducting fluent conversations, and efficiently retrieving information. This technology is acknowledged for its significant advances in natural language processing, benefiting various fields such as intelligent assistants and text creation. On the other hand, negative feedback may suggest that ChatGPT produces incorrect outputs, raises ethical concerns, and has the potential to spread misinformation. These diverse viewpoints reflect the complexity of ChatGPT and its social implications.

### 4.5 Sentiment trend analysis

**Fig 8:**
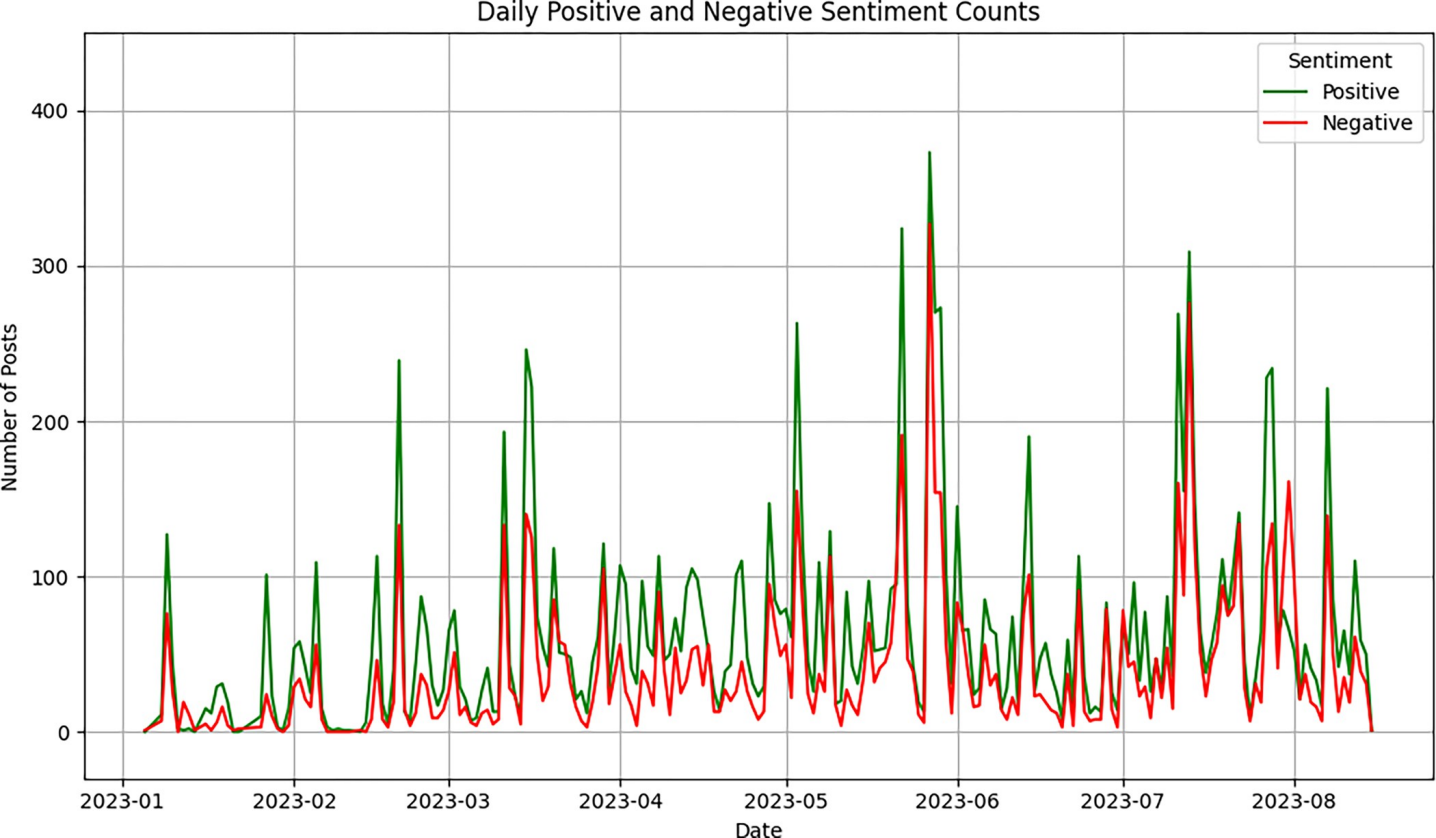
Daily positive and negative sentiment counts.

This part examines daily sentiment trends by comparing the quantity of positive and negative sentiment posts from January to August 2023 (N = 23,773) to explore research question 3. Based on the GPT-3.5 model, ChatGPT was launched by Open AI on November 30, 2022, gaining a growing user base. On March 15, 2023, OpenAI unveiled the new multimodal model, GPT-4, available for purchase [2]. It aims to ascertain whether version updates have influenced sentiment towards ChatGPT. Fig 8 demonstrates how sentiments changed over time. The graph displays two sentiment classes, denoted by green and red, representing positive and negative sentiments, respectively. The sentiments fluctuate over time during the ChatGPT update.

Fig 8 shows that fluctuations in 23,773 entries occur during mid-February, mid-to-late March, May, and mid-July. In most instances, the number of positive entries surpasses that of negative ones. The graph reveals a moderately increasing trend in mid-February, where the daily entry count exceeded 350. This surge can be attributed to the launch of the Plus plan on February 9, offering Plus users the option to select from various versions of ChatGPT. Moreover, the worldwide release of ChatGPT Plus for purchase was announced on February 13th. In mid-March, there was a small peak in the count of entries, approaching nearly 400 daily, perhaps attributable to the announcement of GPT-4 on March 14, 2023. Additionally, there were 100 more positive entries than negative ones indicating that GPT-4’s launch offered advanced reasoning, complex instructions, and enhanced creativity. In May, discussions regarding ChatGPT reached a peak. This surge in activity can be attributed to several factors. Primarily, it could be due to OpenAI’s implementation of new privacy features on May 3rd, which introduced the option to "Turn off chat history and decline to use for model training." It addresses some privacy concerns and encourages user engagement. Furthermore, on May 12th, OpenAI released an update allowing ChatGPT Plus members to incorporate the Bing search engine for browsing web content. It transcended the previous limitations of ChatGPT’s database, which had been confined to information available only until 2021. Lastly, it could also be largely attributed to the momentous launch of the ChatGPT iOS app on May 18th in selected countries, including the United States, the United Kingdom, France, and others. It enriches users’ mobile experiences. However, due to the significant limitations imposed by these successive updates on the nation and the mobile operating system, most users have been unable to benefit from the conveniences. This has potentially led to public dissatisfaction, resulting in a surge of more than 300 negative comments and posts in a single day.

In mid-July, there was a slight increase in ChatGPT discussions, with more than 300 entries reflecting positivity. The surge of positive entries can be attributed to the widespread introduction of the Code Interpreter feature to all ChatGPT Plus users. This innovation allowed non-programmers to express intentions in everyday language, translating into executable Python code solutions, enabling the accomplishment of intricate tasks within a real-time working environment. The innovation not only streamlined the processes of code composition and data manipulation but also expedited the application of artificial intelligence across diverse domains. While the version updates of ChatGPT may spark heated discussions among Reddit users, users generally hold positive sentiments towards ChatGPT and there was no significant shift from positive to negative attitudes during the period between January 2023 and August 2023.

## 5 Discussion

ChatGPT is one of the most fascinating frontier AI technologies, revolutionizing the approach to human-machine interaction and gaining worldwide attention for providing detailed answers in various areas of human society. However, there is an absence of studies evaluating its significant social influence. This study investigates the public’s viewpoints regarding the usage and impact of ChatGPT through topic modeling and sentiment analysis. Differing from sampling survey methods, this study follows the emerging trend of big data mining and gathers data on posts and comments from social media platform, Reddit. It employs the LDA unsupervised learning model to generate seven topics. The study uses a weighted approach that combines VADER with Textblob to categorize sentiment and analyze sentiment trends in posts and comments.

The result reveals seven topics of public discourse concerning ChatGPT, which can be classified into three themes: user perception, technical methods, and impacts on society. It suggests a comprehensive exploration by users into its potential ramifications, with opportunities for advancement across various facets of human society, such as markets, capital, employment, education, research, healthcare, art, entertainment, politics, gender, and ethical considerations. Meanwhile, the extensive discourse on its technical methods indicates that ChatGPT does not replace human intelligence or hinder creative expression. On the contrary, it provides a reservoir of diverse perspectives, facilitating unconventional thinking, and fostering an environment conducive to the expansion of human creative capacities [90, 91].

In addition, sentiment analysis shows that people generally have a positive attitude towards ChatGPT. They believe that ChatGPT can engage in natural and easy conversations with users without requiring an in-depth understanding of complex natural language processing techniques. It is considered a symbol of huge technological progress. However, posts and comments still express concern and criticism about potential risks with ChatGPT. While there are acknowledged limitations within ChatGPT, this study does not explicitly pinpoint the specific areas where these problems exist. Finally, the sentiment analysis reveals that throughout the majority of the periods investigated in our study, most users express a positive attitude towards ChatGPT. Changes in sentiment tend to vary over time and may be affected by updates introduced to ChatGPT. These updates are often associated with a high level of user satisfaction on Reddit.

For practical implication, this study offers valuable insights into potential enhancements and optimal utilization strategies for developers and users of ChatGPT. GPT-related companies and developers should prioritize the user experience. While the public’s attitude towards it is relatively positive due to its naturalistic interactive capabilities, a substantial portion of public discourse (as one of the themes) concentrates on the technical methods of using ChatGPT and its prompts. Therefore, it is recommended that ChatGPT developers enhance the user-friendliness of bot features in product design and its prompt. Additionally, GPT-related companies and research institutions could consider prompt in-depth discussions on technological applications and impacts on society to attract more users. The application of ChatGPT in various fields, such as healthcare, art, and science, can encourage users to unlock the potential of ChatGPT. It promotes cross-domain integration and fosters innovation, even for those with limited knowledge of artificial intelligence techniques or programming [92]. Furthermore, by actively seeking dialogue from diverse stakeholders, this inclusive approach facilitates the ethical development and deployment of ChatGPT.

For the users, they should understand the impact of ChatGPT on their own lives and learn how to use it effectively. The general public needs to learn how to use suitable prompts for text generation and dialogue accurately. Also, users should consider the advantages and disadvantages of ChatGPT. Similar to the findings revealed by previous research [93], the public also expresses concerns about the ethical risks associated with ChatGPT, such as the potential for generating fabricated misinformation, violating copyrights, and promoting plagiarism. Therefore, all stakeholders are expected to cultivate social awareness and engage in public discourse regarding the ethical use and standards of technology. It is crucial to enhance the transparency, accountability, and fairness of ChatGPT [94].

Despite its contributions, this study has several limitations. First, it relies on data from a single social media platform, Reddit, where the users’ demographic skews towards being male, young, white, and highly educated (63% of Reddit users have a Bachelor’s degree or higher) [12, 57]. Previous research indicates that individuals with higher educational attainment and younger age groups exhibit a greater understanding of ChatGPT. This may raise concerns about the generalizability of the findings to users of other social media platforms and the public [95]. Future research should examine the public’s attitude towards ChatGPT on various social media platforms to address the limitation. Comparative analyses across different platforms such as Twitter, Facebook, and online forums would provide a more comprehensive view and public perceptions of ChatGPT. Second, the study is descriptive, and future research should consider causal studies. The study shows a wide range of impacts of ChatGPT on different domains of human society (e.g., market, capital, employment, health, arts, entertainment, politics, and gender). However, it is uncertain whether users with different occupations and identities affect people’s attitudes toward ChatGPT. For example, quantitative methods such as regression analysis can be used. In addition, a longitudinal research design could explore how ChatGPT affects different domains over time. Third, this study does not identify the specific areas in which people expressed negative perceptions. A more detailed qualitative content analysis could examine negative posts and comments to identify specific themes and underlying concerns. This can lead to a better understanding of the limitations of the technology and directions for improvement.

## Supporting information

S1 File Details on data collection and analysis. (DOC)
