# Supplementary material for: The public attitude towards ChatGPT on reddit: A study based on unsupervised learning from sentiment analysis and topic modeling
Source: PLoS One. 2024 May 14;19(5):e0302502. doi: 10.1371/journal.pone.0302502 (PMC11093324; doi:10.1371/journal.pone.0302502)
Supplement: S1 File — (DOC) [file pone.0302502.s001.doc]

# Details on the Study Design

## Dataset Extraction Procedure and Composition

All data used to compile the dataset was downloaded via Apify dump on August 15, 2023. See [https://apify.com](https://apify.com/) for more details. The extraction and cleaning of posts and comments in Reddit was mainly performed using the NLTK tool in Python. See <https://github.com/Shawnzxxu/The-public-attitude-towards-ChatGPT-on-Reddit> for more details. All complete data, as well as processed data, have been collated and can be downloaded manually without re-crawling. They are available through our online appendix. Among them, G3.json, G35.json and G4.json are the unprocessed raw data crawled about the keywords GPT 3.0, GPT 3.5 and GPT 4.0, and Processed_GPT_total.json is the total data after the three versions are processed.

The Reddit post and comment text involves data on user-generated content. Considering data privacy and ethics, we anonymize the user names in the data to protect user identity information. Reddit states in its privacy policy terms <https://www.reddit.com/policies/privacy-policy?rdt=64144>: Third parties are allowed to access public Reddit content through the Reddit API and other similar technologies, and therefore, all data collection and sharing is Complies with Reddit's terms and conditions.
